# Transformative roles of digital twins from drug discovery to continuous manufacturing: pharmaceutical and biopharmaceutical perspectives

**DOI:** 10.1016/j.ijpx.2025.100409

**Published:** 2025-09-27

**Authors:** Ravi Maharjan, Nam Ah Kim, Ki Hyun Kim, Seong Hoon Jeong

**Affiliations:** a College of Pharmacy, Yonsei University, Incheon 21983, Republic of Korea; b College of Pharmacy, Mokpo National University, Jeonnam 58554, Republic of Korea; c Department of Biomedicine, Health & Life Convergence Sciences, BK21 Four, Biomedical and Healthcare Research Institute, Mokpo National University, Jeonnam 58554, Republic of Korea

**Keywords:** Digital Twins, Artificial intelligence machine learning, Real-time monitoring, Continuous manufacturing, Personalized medicine, Dark factory, Automation

## Abstract

Digital Twins (DTs) represent a groundbreaking development tool in the pharmaceutical and biopharmaceutical industries, providing virtual representations of physical entities, processes, or systems. This review investigates the transformative roles of DTs by examining their applications throughout the entire drug development lifecycle, from discovery to continuous manufacturing. By facilitating real-time monitoring and predictive analytics, DTs enhance operational efficiency, reduce costs, and improve product quality. Integration with advanced technologies, such as artificial intelligence and machine learning, further amplifies their capabilities, enabling sophisticated data analysis for preventive maintenance and manufacturing optimization. Despite these advantages, the implementation of DTs faces significant challenges, including data integration, model accuracy, and regulatory complexity. This review discusses these barriers while highlighting opportunities for innovation and automation through emerging technologies, including blockchain, nanotechnology, and dark factory. It also explores the potential of DTs to support personalized medicine through individualized treatments based on patient-specific data. Overall, this review highlights the current state, key challenges, and future perspectives of DT applications in pharmaceutical systems, emphasizing their potential to improve efficiency, quality, and patient outcomes.

## Highlights

• Digital Twins (DTs) transform drug discovery, manufacturing, and clinical trials.
• DTs are instrumental to realize smart manufacture, Industry 5.0, and dark factory.
• DTs can be used in personalized medicine and additive manufacture.
• DTs can save time, resources, and energy.
• DTs can handle all tasks—assembly, inspection, logistics, eliminating human need.

## Graphical abstract

**Figure:**
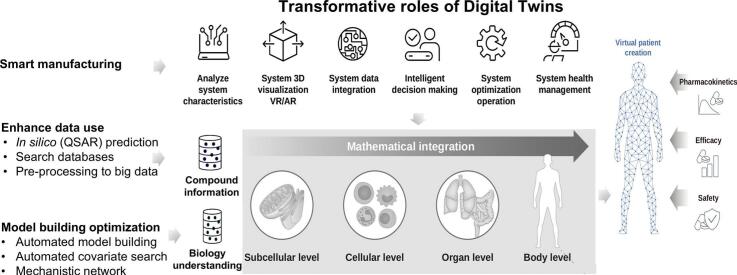

## 1 Introduction

The pharmaceutical industry faces unprecedented challenges: 96 % drug candidate attrition rates (Kumar and Roy, 2025), $2.6B average development costs (Hanchard, 2025), and growing demand for personalized therapies (Gerb and Anggil, 2025; Maharjan et al., 2023b). Digital Twins (DTs) - virtual replicas that link physical systems and computational models through a continuous, bidirectional flow of data - offer transformative solutions through (a) precision discovery (AlphaFold3 showing the potential to power protein-ligand DTs that could reduce target validation time from months to days) (Childs et al., 2025), (b) smart manufacturing (process analytical technology (PAT)-integrated continuous manufacturing DTs improving API consistency to 99.95 %) (Pickles, 2024), and (c) personalized medicine (patient-specific DTs predicting optimal dosages within 7 % of clinical outcomes) (Benedictis et al., 2023; He and Bai, 2021; Lu et al., 2020). Industry 4.0 has ushered in a new era of pharmaceutical development by integrating advanced technologies based on Artificial Intelligence (AI) and Internet of Things (IoT) (Attaran et al., 2023). Originally pioneered by Grieves et al. in 2002 and later expanded by Tuegel et al. in 2011 (Grieves, 2019; Tuegel et al., 2011), DTs establish a dynamic interaction between physical and virtual workshops. The potential applications of DTs in various sectors have been explored in recent years, where six key principles and 127 applications or conceptual proposals in 18 different therapeutic areas are identified (Qi et al., 2021). Several studies have discussed the collaboration of DTs with model construction, data management, and modeling, demonstrating 30–45 % reduction in development timeline and 60–80 % improvement in manufacturing yield (Liu et al., 2021a; Tao et al., 2020).

**Fig. 1:**
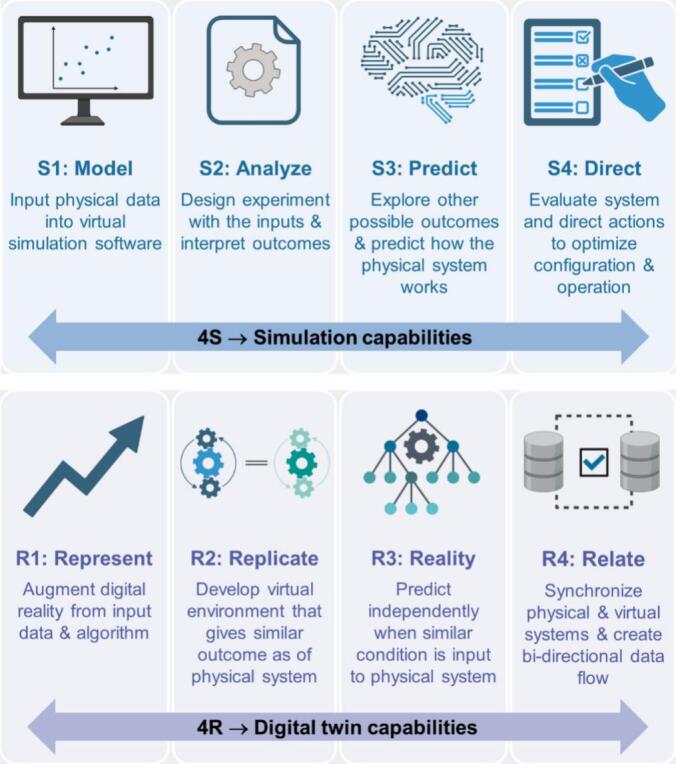
Comparative evaluation of Digital Twins (represent → replicate → reality → relate; bottom) with simulation (model → analyze → predict → direct; top) capabilities, redrawn from (Wooley et al., 2023) with permission from Elsevier.

DTs integrate large volumes of data, AI, and machine learning (ML) to explore new scientific opportunities in the pharmaceutical development, engineering, management, monitoring, analytics, preventive maintenance, forecast, and simulation (Table 1) (Ahmed et al., 2024; Aivaliotis et al., 2019; Billey and Wuest, 2024; Blanco et al., 2024; Brosinsky et al., 2024; Castelló-Pedrero et al., 2024; Duran and Chaudhuri, 2024; Esfandi et al., 2024; Galvão et al., 2024; Gil et al., 2024; Goshisht, 2024; Guerra et al., 2024; Guidani et al., 2024; Gupta and Khang, 2024; Hoang and Nguyen, 2024; Liu et al., 2024a; Maraveas et al., 2024; Mishra and Chakraborty, 2024; Palensky et al., 2024; Prakash et al., 2024; Queipo et al., 2024; Sarwatt et al., 2024; Shariatpour et al., 2024; Subasi and Subasi, 2024; Trayanova and Prakosa, 2024; Umutoni and Samadi, 2024; Walmsley et al., 2024; Wu et al., 2024b). Recent cross-domain applications have expanded to the commercial scale, with prominent discussions on conceptual frameworks and applications for major industry products (Schultes et al., 2022), including Moderna mRNA vaccines, Roche biologics and Siemens medical implants. Compared to conventional simulations, DTs (represent → replicate → reality → relate) enable dynamic optimization and feedback, offering distinct advantages (Fig. 1) (Wooley et al., 2023). They have also proven their utility across diverse industries, such as construction, vehicles, oil and gas exploration, sports performance analyses, telecommunications, and environmental monitoring (Table 1) (AbdElSalam et al., 2024; Abonyi et al., 2024; Ainan et al., 2024; Akram et al., 2024; Al-Shareeda et al., 2024; Bai et al., 2024; Bellavista and Di Modica, 2024; Borges et al., 2024; Dornowski et al., 2024; Gao et al., 2024; Guo et al., 2024; Hartley et al., 2024; Hong and Pula, 2024; Issah et al., 2024; Jeremiah et al., 2024; Joshi et al., 2024; Koo and Yoon, 2024; Letkovský et al., 2024; Li et al., 2024; Long et al., 2024; Lu et al., 2024; Makhoul et al., 2024; Mujawar et al., 2024; Nardini and Stea, 2024; Peng et al., 2024; Qi and Tao, 2018; Rajendiran et al., 2024; Teixeira Filho et al., 2024; Ubuara et al., 2024; Vijouyeh et al., 2024; Wang and Gou, 2024; Wei et al., 2024; Wu et al., 2024a; Yang, 2024; Yousefmarzi et al., 2024; Zhao et al., 2024a; Zhao et al., 2024b). The published review articles related to DTs in the past decades are listed in Table 2.

**Table 1:** The implementation of DTs in different sectors, AI/ML tools used, and the respective advantages in the field.

No.	Sector	AI/ML tool	Application	Reference
1.	Aerospace engineering	Simulate aircraft	Simulate aircraft component	(Mishra and Chakraborty, 2024), (Gil et al., 2024), (Sarwatt et al., 2024), (Castelló-Pedrero et al., 2024)
2.	Agricultural monitoring	Optimize farming	Model farm condition	(Maraveas et al., 2024), (Umutoni and Samadi, 2024), (Galvão et al., 2024)
3.	Autonomous vehicles	Simulate driving scenarios	Simulate vehicle behavior	(AbdElSalam et al., 2024), (Bai et al., 2024), (Borges et al., 2024)
4.	Building management	Optimize building operation	Model building system optimization	(Long et al., 2024), (Li et al., 2024), (Koo and Yoon, 2024)
5.	Energy management	Optimize energy system	Simulate energy usage optimization	(Palensky et al., 2024), (Wu et al., 2024b), (Brosinsky et al., 2024)
6.	Environmental monitoring	Analyze environmental data	Model environmental condition	(Wang and Gou, 2024), (Issah et al., 2024), (Peng et al., 2024), (Guo et al., 2024)
7.	Financial risk management	Predict market trends	Model financial system	(Lu et al., 2024), (Letkovský et al., 2024), (Zhao et al., 2024a), (Ainan et al., 2024)
8.	Fleet management	Optimize fleet operation	Model vehicle behavior	(Joshi et al., 2024), (Zhao et al., 2024b)
9.	Healthcare simulation	Personalized treatment	Simulate patient condition	(Subasi and Subasi, 2024), (Trayanova and Prakosa, 2024), (Queipo et al., 2024)
10.	Manufacturing	Optimize production	Optimize factory process	(Anaya et al., 2024), (Billey and Wuest, 2024), (Walmsley et al., 2024)
11.	Oil/gas exploration	Optimize drilling	Model oil field	(Akram et al., 2024), (Vijouyeh et al., 2024), (Ubuara et al., 2024)
12.	Pharmaceutical development	Optimize drug development	Simulate biological system	(Hoang and Nguyen, 2024), (Duran and Chaudhuri, 2024), (Goshisht, 2024), (Liu et al., 2024a)
13.	Predictive maintenance	Predict equipment failure	Virtual replica for predictive analysis	(Attaran et al., 2024), (Shen and Li, 2024), (Anaya et al., 2024), (Guerra et al., 2024)
14.	Retail analytics	Optimize retail operation	Model store environment	(Huang et al., 2024), (Bellavista and Di Modica, 2024), (Abonyi et al., 2024), (Makhoul et al., 2024)
15.	Smart city	Optimize urban system	Model urban infrastructure	(Shariatpour et al., 2024), (Esfandi et al., 2024), (Prakash et al., 2024), (Blanco et al., 2024)
16.	Sports performance	Optimize training	Model athlete condition	(Yang, 2024), (Dornowski et al., 2024), (Hartley et al., 2024), (Teixeira Filho et al., 2024)
17.	Supply chain management	Optimize planning	Optimize supply chain	(Ahmed et al., 2024), (Gupta and Khang, 2024), (Huang et al., 2024), (Guidani et al., 2024)
18.	Telecommunications	Optimize network usage	Model network infrastructure	(Nardini and Stea, 2024), (Wei et al., 2024), (Jeremiah et al., 2024), (Al-Shareeda et al., 2024)
19.	Water management	Optimize water distribution	Model water system optimization	(Yousefmarzi et al., 2024), (Mujawar et al., 2024), (Hong and Pula, 2024)
20.	Weather forecast	Predict weather pattern	Model atmospheric condition	(Rajendiran et al., 2024), (Wu et al., 2024a), (Gao et al., 2024)

**Table 2:** List of published articles related to DTs along with the number of years evaluated in the study, and year of publication.

No.	Title	No. of years evaluated	Year of publication	References
1.	A comprehensive review of Digital Twin from the perspective of total process: Data, models, networks, and applications	2018–2023	2023	(Wu et al., 2023)
2.	A review of Digital Twin-driven machining: From digitization to intellectualization	2013–2022	2023	(Liu et al., 2023b)
3.	A review of unit level Digital Twin applications in the manufacturing industry	2017–2021	2023	(Böttjer et al., 2023)
4.	A systematic review of Digital Twin about physical entities, virtual models, twin data, and applications	2017–2022	2023	(Liu et al., 2023d)
5.	Bibliometric analysis of Digital Twin literature: a review of influencing factors and conceptual structure	2014–2021	2022	(Wang et al., 2024b)
6.	Characterizing the Digital Twin: A systematic literature review	2009–2018	2020	(Jones et al., 2020)
7.	Digital Twin: Current research trends and future directions	2017–2021	2023	(Alnowaiser and Ahmed, 2023)
8.	Digital Twin-enabled smart facility management: A bibliometric review	2012–2022	2023	(Hakimi et al., 2023)
9.	Digital Twin for smart manufacturing, A review	2013–2023	2023	(Soori et al., 2023)
10.	Digital Twin paradigm: A systematic literature review	2002–2020	2021	(Semeraro et al., 2021)
11.	Digital Twins: Review and challenges	2010–2020	2021	(Juarez et al., 2021)
12.	Digital Twins: State-of-the-art theory and practice, challenges, and open research questions	2009–2021	2022	(Sharma et al., 2022)
13.	Digital Twin technology—A bibliometric study of top research articles based on local citation score	2002–2022	2022	(Ji et al., 2022)
14.	Human Digital Twin in the context of Industry 5.0	2017–2022	2024	(Wang et al., 2024a)
15.	The progress and trend of Digital Twin research over the last 20 years: A bibliometrics-based visualization analysis	2003–2023	2024	(Sun et al., 2024)
16.	Industry application of Digital Twin: from concept to implementation	2005–2021	2022	(Fang et al., 2022)

**Fig. 2:**
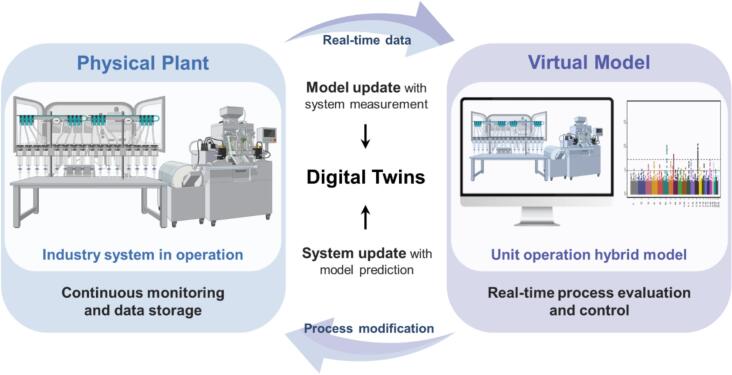
Digital Twin framework in pharmaceutical manufacturing, depicting the bidirectional loop between the physical plant and the virtual model. Real-time data from the physical system update the virtual model, while optimized control commands from the model support process evaluation, modification, and continuous improvement.

**Fig. 3:**
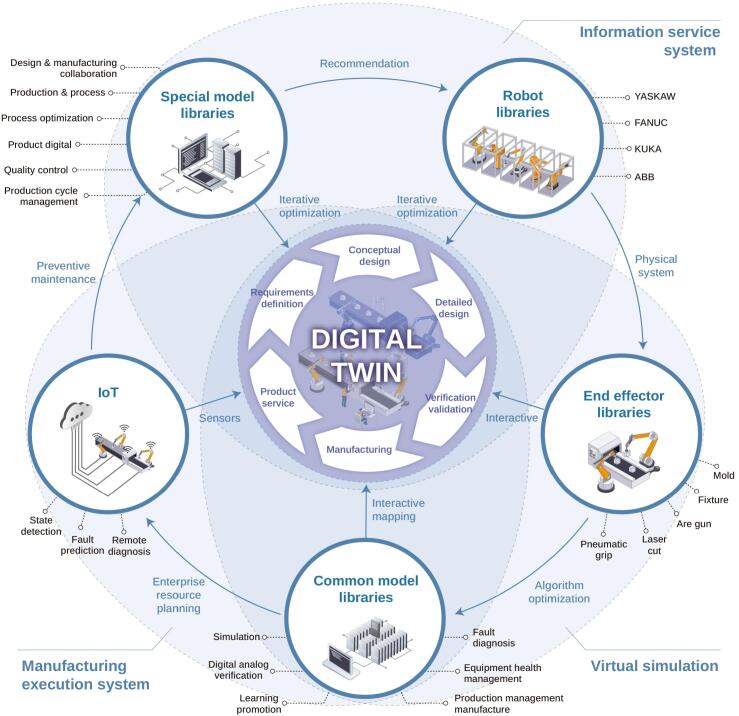
Interactive mapping of conceptual design, details, verification/validation, manufacturing, product service, requirements, and optimization of a Digital Twin model (Robot libraries → End effector libraries → Common model libraries → IoT → Special model libraries) based on information service system, virtual simulation, and manufacturing execution system.

The utilization of DTs with cyber-physical systems (CPS) allows a seamless exchange between physical plants and their virtual models, where real-time data feed and feedback control enable predictive analytics, process optimization, and data-driven decision making (Fig. 2) (Guo et al., 2020; Rasheed et al., 2020; Zakoldaev et al., 2019). Advances in AI, ML, IoT, cloud computing, and data analytics significantly contributed to the progress of DTs framework (Bányai, 2022; Ebni et al., 2023; Kobayashi and Alam, 2024; Rathore et al., 2021; Wang et al., 2022; Zhang et al., 2024). Fig. 3 shows the interactive mapping of conceptual design, details, verification/validation, manufacturing, product service, requirements and optimization of a DT-based on information service system, simulation, and manufacturing execution system (Yu-ming et al., 2020). DTs provide high efficiency and resilience by continuous monitoring, reducing operating and maintenance costs, improving performance, and enabling precise fault diagnosis (Coito et al., 2022; Liu et al., 2023a; Liu et al., 2024b; Shang et al., 2024; Song et al., 2023; Vachálek et al., 2017; Wang and Wang, 2024; Xu et al., 2024; Yang et al., 2023b).

Main components for DTs are physical and virtual ones: a physical component includes all physical data sources such as sensors and network equipment (Bakhshandeh et al., 2024), while the virtual element functions as a digital representation, continuously refining its predictions based on prior knowledge and real-time data (Ren and Zhao, 2023). This dynamic feedback loop ensures precise control over manufacturing processes and supports advanced data analysis. (de Giorgio et al., 2023; Kang and Mo, 2024; Li et al., 2023a). Through robust data management, DTs enhance pharmaceutical manufacturing by providing predictive capabilities, optimization opportunities, and process effectiveness (Alonso et al., 2024; Zhang et al., 2023a; Zhang et al., 2022). Different software has been developed by a number of developers to implement DTs in various fields (Table 3). One of the studies incorporated DTs and sensors in the manufacturing execution systems to control the physical system (Galli et al., 2023; Negri et al., 2020; Shojaeinasab et al., 2022; Vachálek et al., 2017). Another study incorporated platforms like five-dimensional model theories and OpenTwins to boost DTs capabilities through enhanced data visualization, decision-making, and integration of additive technology (Can and Turkmen, 2023; Qiu et al., 2023; Robles et al., 2023; Sun et al., 2020; Wright and Davidson, 2024). Consequently, these developments not onlOy support decentralized smart manufacturing under Industry 4.0 but also align with the resilience concept of Industry 5.0 (Leng et al., 2023; Lv, 2023; Mazumder et al., 2023).

**Table 3:** Different software available in the market for performing DTs, along with their developers, their specific use, categorization of software based on their use, and the software website.

No.	Software	Developer	Use	Category	Website of the company
1.	Ability™	ABB	Industrial platform	IoT	https://global.abb/topic/ability/en
2.	AnyLogic	AnyLogic	Business application	Simulation	www.anylogic.com
3.	Azure DTs	Microsoft	Simulation	IoT	https://azure.microsoft.com/en-us/products/digital-twins
4.	BIM 360	Autodesk	Building information models	BIM	https://www.autodesk.com/bim-360
5.	Digital Sol	DNV GL	Supply chain, product assurance	Analytics	https://www.dnv.com/about
6.	DTs	Emerson Electric	Sensor & optimization	IoT	www.emerson.com/en-us
7.	DTs	Oracle	Predictive maintenance	IoT	https://docs.oracle.com/en/cloud/paas/iot-cloud/iotgs/oracle-iot-digital-twin-implementation.html
8.	DTs	Siemens	Asset performance	IoT	https://www.siemens-advanta.com/capabilities/offers/asset-performance
9.	Forge	Honeywell	Digital transformation	IoT	https://process.honeywell.com/us/en/solutions/honeywell-forge/honeywell-forge-advanced-process-control
10.	Insight	AVEVA	Operation optimization	Analytics	https://www.aveva.com/en/products/insight
11.	Intergraph	Hexagon	Material management	Visualization	https://hexagon.com/products/intergraph-smart-3d
12.	IoT Suite	Bosch	Data analytics	IoT	https://bosch-iot-suite.com
13.	IoT Studio	Altair	Data analytics	IoT	https://altair.com/altair-iot-studio
14.	Leonardo	SAP	Machine learning	IoT	https://mdpgroup.com/en/blog/what-is-sap-leonardo
15.	NX	Siemens	Design and manufacture	Design	https://plm.sw.siemens.com/en-US/nx
16.	Predix	GE Digital	Asset & operations performance	IoT	https://www.ge.com/digital/iiot-platform
17.	Simio	Simio LLC	3D modeling	Simulation	www.simio.com
18.	Simulia	Dassault Systèmes	Finite element analysis	Simulation	www.3ds.com/products-services/simulia/overview
19.	Simulink	MathWorks	Model-based design	Simulation	https://www.mathworks.com/products/simulink.html
20.	Smart manufacture	Rockwell Automation	IoT integration	Automation	https://www.rockwellautomation.com/en-us.html
21.	ThingWorx	PTC	Visualization, AR development	IoT	https://www.ptc.com/en/resources/iiot/product-brief/thingworx-platform
22.	Twin Builder	Ansys	Modeling	Simulation	www.ansys.com/products/platform/twin-builder
23.	Unity Reflect	Unity Technologies	BIM model in virtual reality	Visualization	https://unity.com/pages/unity-reflect
24.	Watson IoT	IBM	Data analytics	IoT	https://www.ibm.com/cloud/internet-of-things
25.	Witness	Lanner	Process modeling	Simulation	https://www.lanner.com/en-gb/technology/witness-simulation-software.html

Despite these advancements, the implementation of DTs in pharmaceutical and biopharmaceutical sectors remains in its initial stages. Distinct challenges in these industries, such as the complexity of integrating diverse data sources, strict regulatory compliance, and the inherent variability of biological systems, have impeded widespread adoption of DTs (Bhujel et al., 2025). Additionally, the absence of standardized protocols and validated models has restricted the scalability of DTs application in this field. Meeting a high standard for process validation, data integrity, and quality assurance requires accurate and dependable DTs models, but many existing frameworks often fall short of these expectations. Furthermore, the complex and multifactorial nature of pharmaceutical systems makes it particularly difficult to develop predictive models that are both adaptable and robust.

The present review discusses the transformative role of DTs across drug discovery, development, clinical trials, and continuous manufacturing. Through the digital representation of molecules and biological systems, DTs are able to identify and accelerate the development of promising drug candidates. The unique preposition that DTs can add to the pharmaceutical science is its ability to contribute toward personalized medicines and increase the survival rates of cancer patients, by tapering the dose based on therapeutic outcomes and drastically reduce the unwanted adverse effects. It is possible with the customization of drug designs and treatment protocols to align individual patient profiles, considering genetic, physiological, and environmental factors. The review also examines their potential to overcome challenges in pharmaceutical and biopharmaceutical manufacturing, highlights successful application, and discusses future perspectives for leveraging DTs to drive innovation.

## 2 Digital Twins in pharmaceutical area

### 2.1 Conceptual framework

**Fig. 4:**
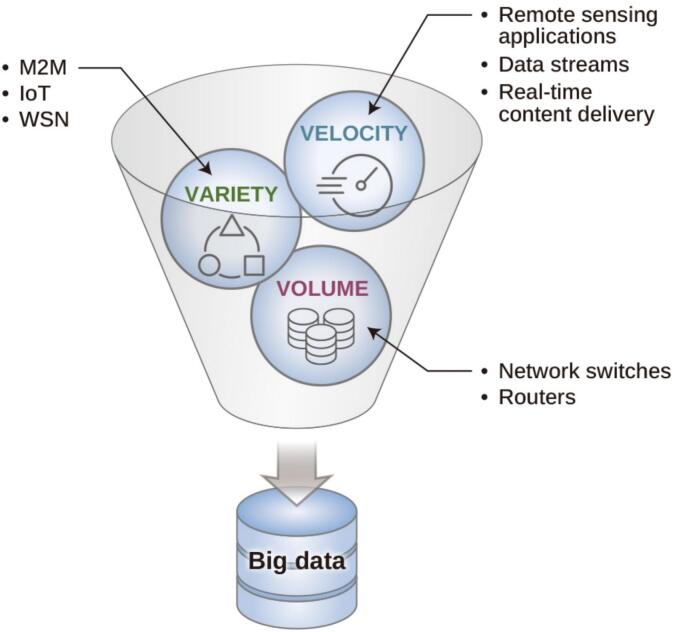
Schematic view of a big data, which consists of volume, velocity, and variety (3Vs). *M2M - Machine-to-Machine communication, WSN - Wireless Sensor Networking Systems.

The pharmaceutical manufacturing sector has incorporated quality-by-design and digitalization to align with the transition toward Industry 4.0 and the emerging Industry 5.0 paradigm. As instrument and process conditions are increasingly digitalized, large and complex datasets are generated, typically described in terms of volume, velocity, and variety, with accuracy and reliability (veracity) being critical for analysis (Fig. 4) (Rathore et al., 2021). This provides the basis for implementing DT applications. Beyond the pharmaceutical context, DTs have also been reported in other industries, including applications in oil and gas and studies on model implementation and limitations, patent issues, and smart manufacturing (Cimino et al., 2019; Wanasinghe et al., 2020). Table S1 summarizes the representative DT application areas relevant to Industry 4.0.

DTs have potential to transform various interdisciplinary fields such as molecular biology, nanotechnology, and pharmaceutical sciences to create a robust delivery system. During the Covid-19 pandemic, it has emphasized lipid nanoparticles (LNPs) that effectively encapsulated oligonucleotides (for example, mRNA and siRNA) to ensure stability and facilitate cellular uptake (Tenchov et al., 2021). This involves optimizing lipid composition and enhancing the bioavailability of the vaccine. The framework adopts advanced characterization techniques to assess the physicochemical properties of LNPs, such as morphology, encapsulation efficiency, particle size, and charge, which are critical for predicting *in vivo* behavior (Szebeni et al., 2023). Lyophilization is another key element that aims to preserve the integrity of oligonucleotides during storage and transport, thereby extending the shelf-life of the products. By integrating these elements, the conceptual framework not only addresses the technical challenges associated with oligonucleotide delivery but also lays the foundation for future innovations in vaccine formulation and manufacturing processes, contributing to the development of effective and accessible therapeutics.

A prior study has established a relationship between DTs and lean Six Sigma strategy (Maheshwari and Devi, 2024). Currently, there is no universally accepted technical DTs standard. To address the issue, the JTC1 advisory group on emerging technology innovation has developed a protocol and defined in ISO 23247:2021 as “fit-for-purpose digital illustration of a manufacturing component to converge the component with its illustration at suitable synchronization rate” (Lei et al., 2021; Shao et al., 2023a; Shao et al., 2023b; Wallner et al., 2023). Technically, other standards such as ISO 10303, ISO 13399, and Open Platform Communications unified architecture provide guidelines for sharing data within systems (Morshedzadeh et al., 2022). They are virtual platforms that mimic the behavior and dynamics of physical systems, allowing real-time monitoring and prediction of system performance (Fuller et al., 2020). Their framework consists of three key factors: physical components, virtual components, and the data exchange between them, which can play a pivotal role in enhancing the quality and efficiency of production processes (Fuller et al., 2020). A previous study has proposed a reference model encompassing three perspectives – hierarchy, dimension, and scale – to promote model reusability, interpretability, and adaptability (Liu et al., 2023c). Another study introduced a spatial DTs that emphasized dimensional attributes (Ali et al., 2023). Despite the advancements, their comprehensive implementation on the pharmaceutical industry is still nascent. The key factors for developing DTs are PAT, modeling, and dataset incorporation in the sector. One study incorporated a DT-based supervised AI/ML model to control the production process (Alexopoulos et al., 2020).

In the aspect of intelligent manufacturing systems, DTs offer exceptional capability to enable real-time understanding and predict potential failures. The resilience of the system is enhanced through their application, which contributes to improved quality, productivity, and flexibility while reducing costs (Fuller et al., 2020). Prior studies emphasize the importance of enabling technologies, such as AI/IoT, in developing DTs (Davidopoulou and Ouranidis, 2022). These technologies facilitate seamless integration of data between physical and virtual systems, which is essential for effective implementation. The prospects presented by the technologies are critical areas of ongoing research. Although many implementations have been successfully achieved, there is still a lack of focused research on DTs application in the pharmaceutical industry (Liu et al., 2021b). This gap emphasizes the need for further investigation into the key components, developments, and applications of DTs in pharmaceutical areas. A previous study has indicated that while the drug manufacturing sector is progressing toward implementing DTs, there are still significant challenges in the foreseeable future (Alnowaiser and Ahmed, 2023). The integration of technologies and robust models are essential for their realization in pharmaceutical manufacturing, which promises to enhance the efficiency, quality, and sustainability of the processes (Chen et al., 2020).

### 2.2 Drug discovery and development

**Fig. 5:**
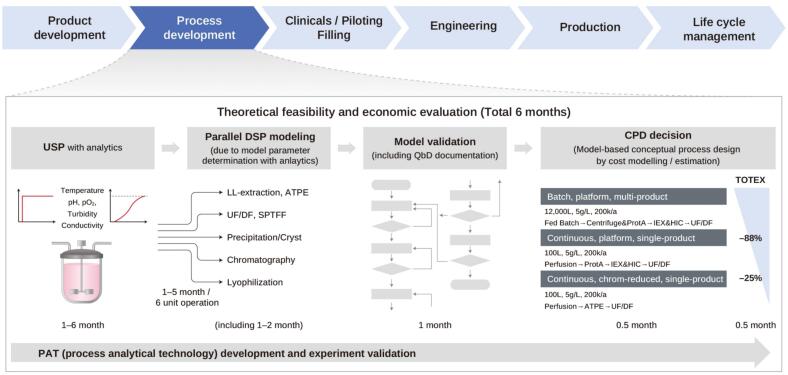
PAT tool incorporated in Digital Twins system (product development → process development → clinical/filling → engineering → production → lifecycle management). For instance, PAT implementation in the process development along with experimental validation are shown to perform theoretical feasibility and economic evaluation of 6 months product cycle.

DTs in pharmaceuticals encompass the virtual replicas of drug molecules, biological systems, and patients (Nye, 2023). During the product development lifecycle (Fig. 5, product development → process development → clinical/filling → engineering → production → lifecycle management), different process technologies can be incorporated in the DTs. For instance, PAT implementation in the process development along with experimental validation have been used to conduct theoretical feasibility and economic evaluation of 6-months product cycle. This enables to simulate and analyze drug behavior, efficacy, and safety in a controlled virtual environment (Fig. 5) (Zobel-Roos et al., 2019). Scientists conduct large *in silico* experiments, which can be too expensive and laborious if in a standard laboratory. For instance, DTs can be used to explore different drug formulations and delivery systems to identify the most promising candidates early in the development process. DTs facilitate the computer-aided digital representation of drug candidates, allowing scientists to predict pharmacokinetics, pharmacodynamics, and safety profiles. This accelerates the identification of lead compounds and reduces the likelihood of late-stage failures, which are often attributed to unforeseen safety issues or a lack of efficacy.

### 2.3 Clinical application and personalized medicine

The advent of DTs represents a transformative approach to personalized medicine, drug discovery, and clinical trials (Turab and Jamil, 2023). DT models can replicate the physical and biological characteristics of a patient or system, enabling simulations to predict outcomes and optimize treatments. This capability is particularly valuable in personalized medicine, where understanding patient-specific genetic and physiological factors can guide treatment strategies. DTs are employed in healthcare for precise diagnosis and personalized treatment, with potential across various medical fields (Kaul et al., 2023). For instance, in cardiology, DTs can model heart function and predict the impact of different interventions such as medications or surgical procedures. Clinical DTs simulate patient outcomes, reduce sample size, and maintain data confidentiality (Chen et al., 2020). In oncology, DTs have been used to model how individual patients may respond to chemotherapy regimens, thereby supporting oncologists in selecting the most appropriate treatment plan (Shen et al., 2025; Sun et al., 2023; Wu et al., 2025). Image-guided digital twins have made it possible to predict patient responses to Doxorubicin/Cyclophosphamide and Paclitaxel (Wu et al., 2025). A biology-based digital twin was further applied to a personalized Doxorubicin/Cyclophosphamide regimen in neoadjuvant breast cancer therapy, enabling dose reduction based on patient-specific response (Christenson et al., 2025). Beyond cancer, DTs have been used in sex-specific quantification of Class III anti-arrhythmic drugs, such as Amiodarone, Dofetilide, and Dronedarone, by integrating *in vitro* ion-channel data with *in silico* models and AI/ML tools (Bai et al., 2025). Additional applications include 3D personalized chips for ischemic stroke patients with on-chip screening of antiplatelets and anticoagulants to reduce thrombotic risk at an early stage (Zhao et al., 2025), and quantitative systems pharmacology (QSP)-based approaches supporting enzyme replacement therapies in Pompe disease with Alglucosidase alfa and Avalglucosidase alfa (Kaddi et al., 2025).

Health DTs simulate human physiology and diseases to improve drug discovery, select targets, design clinical settings, and enable precision therapy and public health interventions (Björnsson et al., 2019). By integrating real-world data from electronic health records, wearable devices, and genomic information, they provide a comprehensive view of patient health and facilitate informed clinical decisions. Generative AI empowers them by designing a complex drug screening approach and realistic clinical trials, although its full potential remains untapped (Venkatesh et al., 2024). By generating virtual patient data that reflects real-world variability, researchers can work on robust simulations and thus improve the degree of model accuracy. AI-driven DTs in healthcare, especially in cancer care, can integrate diverse data to improve clinical decision support and individualized care (Bordukova et al., 2024). By leveraging AI/ML algorithms, they can simulate individual patient responses, streamline the processes, and enhance the precision of treatment decisions (Haleem et al., 2023).

Patient-specific DTs can predict individual responses to viral infections, which aids treatment decisions and pandemic responses (Das et al., 2023). Particularly, this capability is relevant to the emerging infectious diseases, where rapid and accurate predictions can inform public health strategies. Furthermore, the successful application of DTs in manufacturing patient-specific medical devices, such as 3D-printed orthopedic implants, provides a clear example of how personalization technologies are being realized. Together, these applications establish the foundation for the more systematic, quantitative approaches that are further elaborated in the following section on pharmacometrics.

## 3 Integration of pharmacometrics and digital twins

**Fig. 6:**
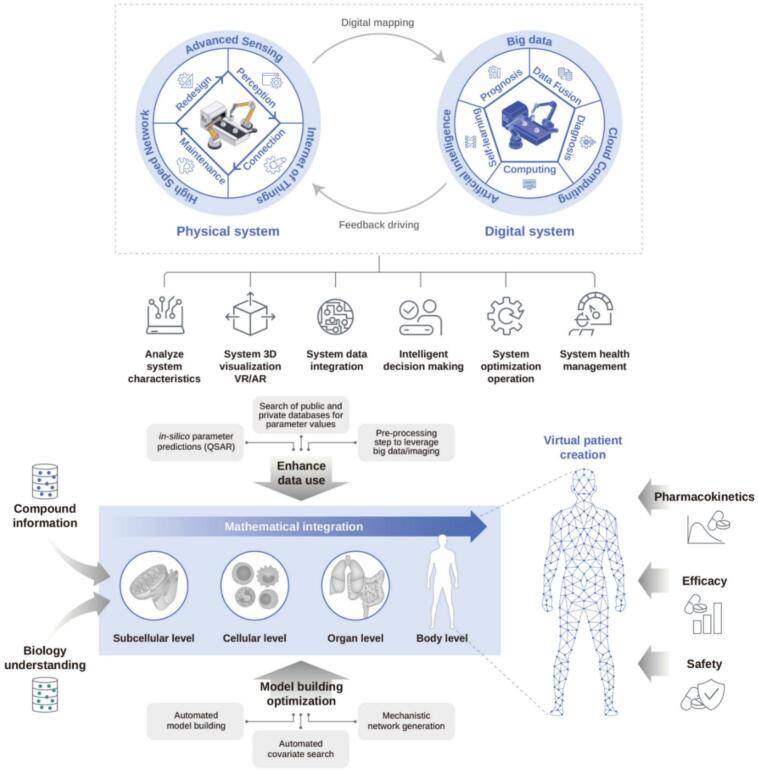
Smart manufacturing with Digital Twins, model building and optimization, and their influence on pharmacokinetics, efficacy, and safety, redrawn from (Soori et al., 2023) under Creative Commons License CC BY 4.0 DEED, permission from Elsevier.

Pharmacometrics is an emerging science that quantifies drug, disease, and trial information to aid efficient drug development and regulatory application. This enables the modeling and simulation of complex drug-response relationships in diverse patient populations. When integrated with DTs, pharmacometric models can enhance exposure–response analyses, optimize dosing strategies, and provide quantitative evidence for regulatory decision-making. By utilizing DTs, researchers can create high-resolution models that accurately reflect individual patient characteristics, allowing for computational testing of thousands of drugs against the models. Smart manufacturing with DTs, model building and optimization, and their influence on pharmacokinetics, efficacy, and safety are illustrated in Fig. 6. This integration allows researchers to move beyond qualitative personalization and into quantitative modeling. Patient-specific avatars, as part of pharmacometric frameworks, provide insights into PK/PD relationships and treatment optimization that are not achievable using conventional methods (Chasseloup et al., 2023).

DTs improve drug discovery, clinical design, precision therapy, and drug repurposing (An and Cockrell, 2022). By mimicking different treatment settings, they can discover the most favorable drug candidates for individual patient populations, thereby rationalizing the process. Additionally, they can enhance the productivity of participants in clinical trials by identifying individuals who are most likely to benefit from a particular treatment based on their exclusive profiles. Moreover, they support model-based system engineering and integrate simulations with IoT to enhance the system methodologies (Booyse et al., 2020). This incorporation creates continually updated virtual replicas of physical systems, distinguishing them from static models (Venkatesh et al., 2024). By leveraging online data and advanced analytics, they facilitate predictive healthcare analytics and personalized patient care, enabling healthcare providers to make an informed decision (Das et al., 2023). Deep DTs take this step further by automating preventive maintenance and health monitoring without exclusively relying on the history of failure data, a capability that is remarkably valuable in healthcare settings where appropriate interventions can significantly improve patient outcomes (Madni et al., 2019). Clinical DTs generated by AI/ML models can lower the need for massive participant recruitment while enhancing data-sharing privacy. From a regulatory pharmacometrics perspective, DTs can complement population PK modeling and quantitative systems pharmacology, offering more robust evidence to support dose justification. This is uniquely important in an era where patient privacy is utmost important, and the ethical handling of sensitive health data is critical. A modular computational framework for medical DTs allows for the efficient integration of heterogeneous models, thereby improving scalability and adaptability (Wright and Davidson, 2020). This flexibility is essential in a rapidly growing healthcare setting, where new data sources and technologies are continually emerging. By accommodating various data types and modeling approaches, they provide a comprehensive view of patient health and facilitate personalized and effective treatment strategies.

## 4 Biopharmaceuticals

### 4.1 Modeling of biopharmaceutical system

Modeling biopharmaceutical systems is inherently complex, with challenges ranging from improving the bioavailability of active ingredients to managing the variability of cell culture processes. For instance, to address solubility-related issues, DTs have been employed to optimize the manufacturing process of nanosuspensions (Sokolov et al., 2021). An even more prominent application lies in managing sophisticated biologics production, as highlighted by a detailed case study. For instance, Novartis reported a 22 % batch failure rate in its monoclonal antibodies (mAbs) production line, primarily due to unpredictable cell metabolism shifts and inefficient harvest timing decisions (Simpson, 2011). The existing system relied on offline glucose and lactate measurements taken at 6-h intervals, along with legacy PAT from 2015, resulting in delayed parameter adjustments. To address these limitations, DT-driven optimization is implemented, which requires a deep, model-based understanding of the process. As a foundational step for mAbs production in fed-batch Chinese hamster ovary (CHO) cell culture, a robust design space was established by using design of experiments and multiple linear regression analysis to identify the optimal set points for critical process parameters (Wohlenberg et al., 2022). Building upon such foundational process models, a more advanced hybrid AI framework can be developed to simulate CHO cell growth dynamics across genomic, metabolomic, and bioreactor levels. This advanced DT approach has enabled the prediction of optimal harvest window within ±15 min accuracy across 92 consecutive batches, real-time monitoring of 47 bioreactors with cross-facility synchronization and has reduced technology transfer time for new mAbs from 18 to 6.5 months. The benefits of the DT implementation are summarized in Table 4.

**Table 4:** Operational Outcomes of the case study.

Metric	Pre-DT (2023)	Post-DT (2025)	Regulatory Impact
Batch success rate	78 %	94 %	Reduced OOS events by 83 %
Titer variability	±18 %	±6 %	Enabled real-time release (RTR)
Media consumption	12,400 L/batch	9800 L/batch	$28 M/year cost savings
Time-to-critical decision	5.3 h	8.6 s	99.7 % faster corrective actions

Despite their considerable potential, the realization of DTs in biopharmaceutical manufacturing faces significant practical challenges, as highlighted by the case study. A key technical hurdle was bridging legacy process control systems with modern DT architecture, while on the modeling front, the DT struggled to generalize from historical data to a novel cell line. The most significant barrier is regulatory alignment, demonstrated by the 9-month validation period required to meet FDA's 21 CFR requirements for data integrity, traceability, and audit readiness in manufacturing records. (Lin et al., 2022; Ramadan et al., 2021). These issues underscore the need for advanced approaches, such as hybrid models, to manage the inherent heterogeneity and uncertainty of biopharmaceutical processes (Tao et al., 2018). In biopharmaceutical settings, a key challenge lies in integrating diverse elements, including modeling, lifecycle management, and interdisciplinary collaboration, into a cohesive framework (Sokolov et al., 2021). Hybrid DT models address this need by providing a central platform for such integration, which allows information from different domains to be connected and analyzed in real time. A key benefit of this is improved fault diagnosis, since early deviations in process data can only be detected reliably when process models, operational history, and expert knowledge are considered together. Techniques such as support vector machines (SVM), Bayesian networks, and deep learning can then be incorporated into these models to enable timely issue detection, real-time maintenance, and the prevention of irreversible failures. (Amin et al., 2021; Jain et al., 2019; Kohtz et al., 2024; Wang et al., 2019; Wang et al., 2023; Xia et al., 2021; Xia et al., 2023; Yang et al., 2023a; Zhang et al., 2023b; Zhu et al., 2023). From a regulatory perspective, hybrid DT models can support alignment by accelerating process understanding and generating traceable evidence to complement experimental studies. Although they do not currently replace formal validation requirements, such models may reduce redundancy and help mitigate delays that often arise during regulatory review.

**Fig. 7:**
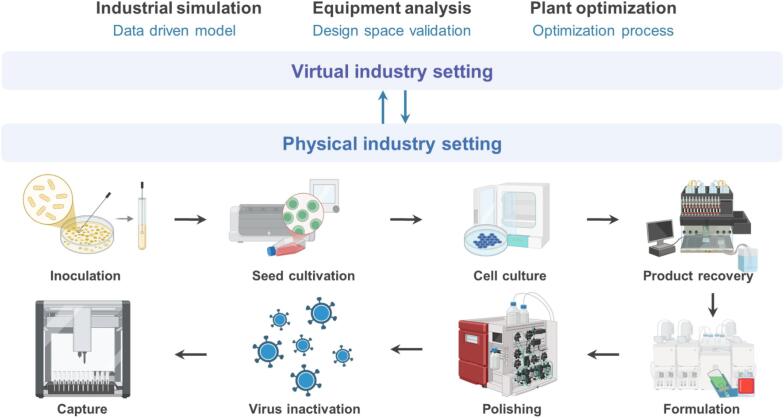
Digital Twins implemented in virtual setup of the industrial simulation, equipment analysis, and plant optimization, which are linked with the physical industry setting (inoculation → seed cultivation → cell culture → product recovery → capture → virus inactivation → polishing → formulation) of a biopharmaceutical process.

However, even with these advancements in process modeling and PAT, the application of DTs in pharmaceutical manufacturing remains in its early stages, highlighting the need for continuous research and development (Fuller et al., 2020). The ability of DTs to construct adaptive, high-fidelity, multidimensional models, as already demonstrated in aerospace component manufacturing, suggests strong potential for similar applications in the biopharmaceutical domain (Canzoneri et al., 2021). Realizing this potential, successfully deployed DTs are used in virtual environments for industrial simulation, equipment analysis, and plant-wide optimization, and are integrated with physical production settings (inoculation → seed cultivation → cell culture → product recovery → capture → virus inactivation → polishing → formulation) of a biopharmaceutical process, as illustrated in Fig. 7. Recognized as a transformative technology, DTs align with the principles of Industry 4.0 by enabling intelligent, responsive, and interconnected biopharmaceutical manufacturing systems (Javaid et al., 2023). DTs replicate the dynamics of physical systems, offering capabilities such as online monitoring, preventive maintenance, and advanced process control. These functions enhance intelligent manufacturing by providing real-time awareness of system states and predicting failures, thereby promoting sustainable production practices (Xu et al., 2019).

### 4.2 Biopharmaceutical production

Beyond the foundational modeling discussed in Section 4.1, DTs are integrated into biopharmaceutical production to apply these advanced frameworks in real-world settings. By creating a virtual representation of the process system, DTs facilitate superior decision making and operational efficiency (Chen et al., 2020). Their major benefits lie in employing real-time data from various sources including sensors and PAT, to enable continuous monitoring of critical quality and process parameters. This allows manufacturers to promptly respond to deviations and optimize production conditions (Maharjan and Jeong, 2022). As a case in point, real-time feedback from DTs can guide adjustments in temperature, pH, and nutrient concentrations during cell culture processes, thereby ensuring optimal growth conditions and product yield (Schmidt et al., 2022). More recently, the hybrid modeling combined with transfer learning has accelerated bioprocess DTs design and improved the predictive accuracy (Riezzo et al., 2025). For instance, a study used a hybrid model to optimize a dynamic perfusion CHO cell culture process *in silico*, which led to a 50 % increase in volumetric productivity. The model also demonstrated reliable extrapolation capabilities to larger 5 L bioreactors, confirming its utility for scale-up (Agarwal et al., 2025).

Although this section focuses on biopharmaceuticals, insights can also be drawn from continuous manufacturing in the small-molecule domain, which provides validated frameworks directly translatable to biologics. A notable case reported that the production of Prezista® transitioned from traditional batch manufacturing to continuous processing. While batch production required seven different rooms and took two weeks, the DT-enabled continuous manufacturing process was consolidated in two rooms and completed in a single day (Gong et al., 2025). This transition resulted in a 33 % reduction in waste and an 80 % reduction in manufacturing and testing cycle time. In-line near-infrared (NIR) spectroscopy further ensured continuous tablet production by monitoring powder homogeneity during transfer to the compression station. This case study provides a clear blueprint for how DTs can enable end-to-end process control and principles which are directly sought in biopharmaceutical continuous processing (Gong et al., 2025).

Beyond process optimization, DTs leverage advanced analytics, including AI/ML algorithms, to predict outcomes based on historical and real-time data. This predictive capability is critical for identifying potential bottlenecks and inefficiencies in manufacturing processes. By simulating various scenarios, manufacturers can optimize workflows, reduce cycle times, and enhance overall productivity (Canzoneri et al., 2024). To illustrate, preventive maintenance enabled by DTs forecasts equipment failures before they occur, thereby minimizing downtime and maintenance costs. DTs have also been implemented across R&D, production, and quality assurance, providing integrated virtual platforms that support informed decision-making and enable cross-disciplinary coordination (Ding et al., 2023).

As regulatory requirements have become increasingly stringent, DTs play a key role in ensuring regulatory compliance. By maintaining a comprehensive digital record of the manufacturing process, they facilitate traceability and accountability. Automated reporting features streamline the documentation process, ensuring that all the necessary data are readily available for regulatory audits (Destro et al., 2023). Looking ahead, future studies could focus on developing standardized frameworks for DT implementation, enhancing data integration, and refining models with AI (Maharjan et al., 2024a). Ethical issues related to data usage and patient privacy must also be addressed (Ding et al., 2023). Overall, DT integration in biopharmaceutical manufacturing not only addresses current challenges but also paves the way for a more dynamic, robust, and efficient production landscape as the industry embraces Industry 4.0.

### 4.3 Advancements in immunogenicity

DTs are implemented to predict immunogenicity in biotherapeutics, offering a transformative approach in this field. By leveraging computational models, they facilitate a deeper understanding of the interactions of the immune system with therapeutic agents, leading to safer and more effective medications (Destro et al., 2021). For example, DTs simulate the pharmacokinetics and pharmacodynamics of proteins to provide insights into how different formulations are processed by the body and elicit immunogenic responses. (Björnsson et al., 2019). AI/ML-enhanced DTs have been applied to evaluate vaccine responses, where microstructural marbling at injection sites was modeled against cell concentration and growth (Zohdi, 2022). Integration of multi-omics data—genomics, proteomics, and metabolomics—further improves immunogenicity prediction, enabling biomarker identification and supporting personalized therapies (Komalasari, 2023). DTs also facilitate *in silico* screening of therapeutic candidates by simulating various drug formulations and their potential immunogenic effects. This process drastically reduces time and resources required for conventional *in vitro*-*in vivo* studies. By predicting the immunogenicity of multiple candidates instantaneously, the researchers can prioritize the most promising options for further development. This efficiency is specifically favorable in the fast-paced biopharmaceutical aspect, where prompt advancements are needed. They can identify genomic signatures associated with immunogenicity, aiding in the selection of therapeutic epitopes (Björnsson et al., 2019). *In vitro* models, including 2D and 3D assays, are used to assess the immunogenicity of therapeutic proteins, which is an important step in drug development (Smith et al., 2019).

DTs play a pivotal role in predicting immunogenicity and optimizing vaccine formulations. By simulating the immune response to different vaccine components, researchers can identify the most effective combinations of antigens and adjuvants. This predictive capability is valuable in the development of personalized vaccines, where individual patient characteristics can be considered to enhance efficacy and minimize adverse reactions (Laubenbacher et al., 2021). There are challenges in translating these models into clinical practice. Concerns such as data consistency, model validation, and regulatory approval must be addressed to facilitate the extensive acceptance of DTs in biopharmaceutical development. Collaborative efforts between academia, industry, and regulatory bodies are essential to establish guidelines and best practices for their implementation in clinical settings. They optimize patient-specific treatment regimen by accounting for uncertainties in tumor biology, potentially improving outcomes in diseases such as high-grade gliomas (Chaudhuri et al., 2023). Patient-specific DTs could integrate human physiology and immunology with real-time clinical data to predict the course of viral infections and immune responses, enhancing treatment effectiveness (Groell et al., 2018). Overall, DTs combined with AI/ML offer strong potential for target selection, treatment optimization, and prediction of disease progression, though significant challenges remain in clinical translation.

The prospective study needs to focus on refining the models to incorporate real-time patient data, enhancing their predictive capabilities (Maharjan et al., 2023a). Furthermore, exploring the integration of DTs with emerging technologies, such as wearable devices and mobile health applications, could provide valuable insights into patient responses and treatment outcomes. By continuously updating them with new data, researchers can improve their accuracy and relevance, leading to better patient care. They represent a powerful tool for predicting immunogenicity in biopharmaceuticals. By simulating complex immune interactions and integrating diverse data sources, DTs can advance drug design, enhance vaccine development, and support personalized medicine. Addressing the challenges of clinical translation will be crucial for realizing their full potential.

## 5 Additive manufacturing

### 5.1 Design flexibility and customization

**Fig. 8:**
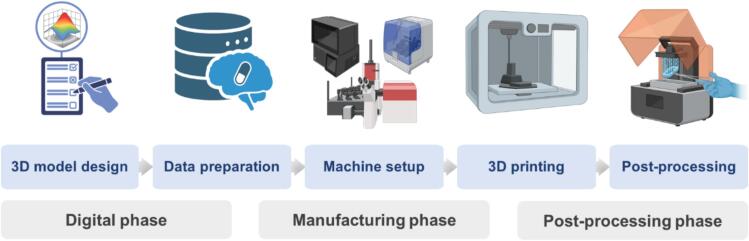
Process flow diagram of an additive manufacturing that includes digital phase, manufacturing phase, and post-processing phase. Process flowchart is in the order of 3D model design → data preparation → machine setup → 3D printing → post-processing, redrawn from (Haw et al., 2022) with permission from Elsevier.

One of the major advantages of integrating DTs into additive manufacturing is enhanced design flexibility. DTs enable rapid prototyping and iterative design processes, allowing manufacturers to explore a wide range of configurations without the constraints of traditional manufacturing methods. Fig. 8 illustrates a flow diagram of the additive manufacturing process, which consists of three main phases: digital phase, manufacturing phase, and post-processing phase. The process sequence includes 3D model design → data preparation → machine setup → 3D printing → post-processing (Haw et al., 2022). This flexibility is particularly valuable in the healthcare industry, where customized components such as patient-specific implants are important. DTs can simulate the performance of various geometries and materials, allowing engineers to optimize designs for individualized applications.

A recent example is Siemens Healthineers' implementation of DTs to address data integration challenges in the production of orthopedic implants (Mihai et al., 2022). The project focused on 3D-printed titanium hip prostheses, where incompatible data formats between legacy AutoCAD software from 1990s and modern selective laser melting printers resulted in 32 % production delays and $1.2 million in annual scrap costs. To resolve these issues, a hybrid integration architecture was introduced. A custom gateway bridged protocol mismatches by translating legacy protocols into IoT compatible formats and reduced data latency from 650 to 8 ms while maintaining strict thermal control of the powder bed 15 ± 2 °C. The solution also incorporated a Python-based file converter that ensured compliance with ISO/ASTM 52915 standards, achieving 99.97 % format integrity. Quality management systems were modernized using blockchain-secured audit trails that unified 57 production data streams into a regulatory cloud system compliant with EU MDR 2017/745. As a result, audit preparation time was reduced from 14 to 2 days, and first-pass yield improved from 68 to 93 %. Post-implementation analysis also showed a 25 % reduction in geometric deviations in curved lattice structures critical for bone ingrowth, and 47 % reduction in lead time (from 17 to 9 days). The implementation has since been replicated across 14 Siemens facilities, demonstrating how DTs can achieve both technical modernization and regulatory compliance in medical additive manufacturing applications without requiring full system replacement. This case provides a crucial insight: successful digital transformation in regulated manufacturing requires a concurrent regulatory strategy, not just a technical one. By strategically using such novel tools for compliance, the implementation serves as a concrete model for ensuring the traceability and reliability of patient-specific data. This approach directly addresses the core ethical and regulatory challenges facing the adoption of personalized manufacturing technologies, offering a viable pathway for other medical device manufacturers.

### 5.2 Real-time monitoring and quality assurance

**Fig. 9:**
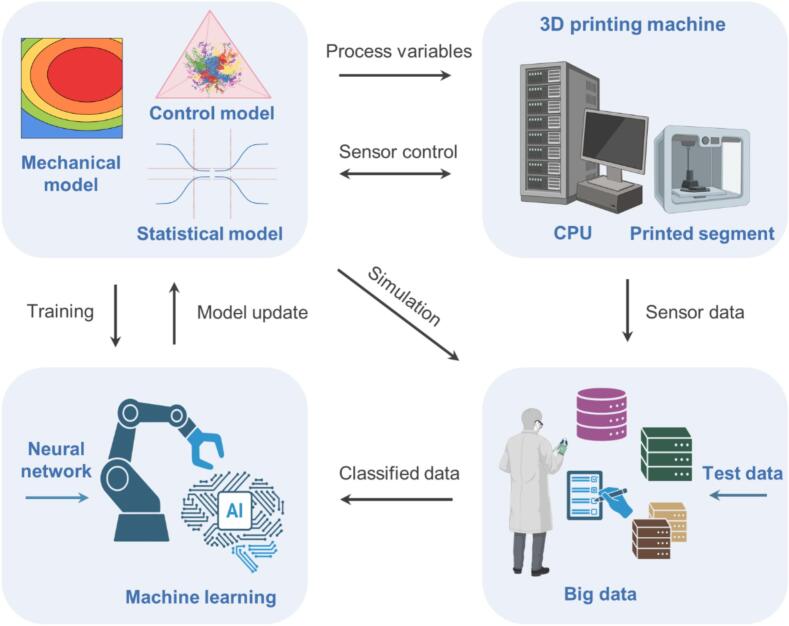
Incorporation of Digital Twins on the additive manufacturing requires four fundamental elements: 3D printing machine, sensor and literature data, machine learning algorithm, and models (mechanical, control, and statistical).

The integration of DTs in additive manufacturing facilitates real-time monitoring of the production process. By continuously collecting data from sensors embedded in the 3D printing equipment, they provide insights into critical parameters such as temperature, pressure, and material flow. This real-time feedback loop enables manufacturers to detect anomalies early in the production process, allowing for immediate corrective actions. Incorporation of DTs on the additive manufacturing requires four fundamental elements: 3D printing machine, sensor and literature data, machine learning algorithm, and models (mechanical, control, and statistical), as illustrated in Fig. 9. They function as a virtual representation of the additive manufacturing process, which enabling the optimization of manufacturing operations and product quality. For example, if a DT identifies a deviation in temperature during the printing of a medical implant, operators can adjust the settings on the fly to prevent defects, ensuring that the final product meets stringent quality standards. The design of DTs for additive manufacturing requires the validation of models that can predict metallurgical parameters influencing the structure and properties of components (DebRoy et al., 2017). They facilitate the design of smart manufacturing systems by enabling hardware-in-the-loop simulations, which can prevent expensive reconfiguration by identifying design deficiencies early (Liu et al., 2019).

### 5.3 Preventive maintenance and operational efficiency

**Fig. 10:**
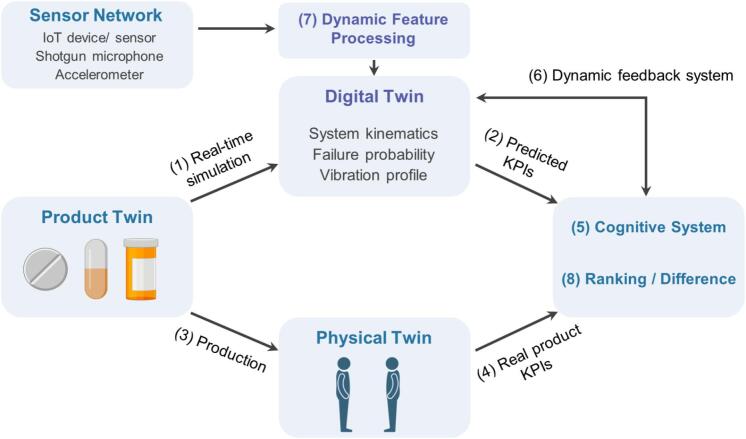
Stepwise implementation of Digital Twins on the additive manufacturing: 1) Real-time simulation, 2) Predicted KPIs, 3) Production, 4) Real product KPIs, 5) Cognitive system, 6) Dynamic feedback system, 7) Dynamic feature processing, and 8) Ranking and difference.

DTs also play a crucial role in preventive maintenance within additive manufacturing systems. By analyzing historical and real-time data, they predict when equipment is likely to fail or require maintenance. This preventive capability minimizes unplanned downtime and extends the lifespan of machinery, leading to increased operational efficiency. For instance, a study demonstrated that by implementing DTs for preventive maintenance in a 3D printing facility, manufacturers could reduce maintenance costs by up to 30 % while improving overall equipment effectiveness. It is achieved through a stepwise implementation of DTs in additive manufacturing, consisting of the following stages: 1) Real-time simulation, 2) Predicted key performance indicators (KPIs), 3) Production, 4) Real product KPIs, 5) Cognitive system, 6) Dynamic feedback system, 7) Dynamic feature processing, and 8) Ranking and difference (Fig. 10).

### 5.4 Integration with Industry 4.0

The integration of DTs with other Industry 4.0 technologies, such as AI/IoT, further enhances the capabilities of additive manufacturing. IoT devices can provide real-time data inputs for DTs, while AI algorithms analyze these data streams to optimize production processes. For example, AI can be used to refine the process parameters of additive manufacturing based on insights generated by DTs, leading to improved product quality and reduced material waste. This integration creates a smart manufacturing ecosystem, in which data flows seamlessly between physical and digital realms, enabling manufacturers to make informed decisions quickly. A prior study implemented DTs to produce medical implants using additive manufacturing, in compliance with ISO 13485:2016 and ISO 14971:2012 standards, ensuring repeatability and reliability of the process (Thejane et al., 2023). High-fidelity models simulating key factors affecting product quality were employed to support the construction of structurally sound parts (Scime et al., 2022). To enable the development and deployment of such models, scalable cyber-physical infrastructures have been proposed, incorporating augmented intelligence to predict component performance (Liu et al., 2021b). DTs are increasingly recognized as a key technology in smart manufacturing, with applications across product design, production, and health management, despite ongoing challenges in their development, integration, and standardization (Kantaros et al., 2022). In additive manufacturing, robust process control systems by DTs help manage uncertainties and maintain consistent performance within predefined limits (Yang and Özel, 2021).

The implementation of DTs in additive manufacturing presents several challenges. One significant hurdle is the need for comprehensive data integration across heterogeneous systems and processes (Knapp et al., 2017). Many manufacturers still rely on legacy systems that may not be compatible with modern digital frameworks, resulting in data silos and operational inefficiencies. Furthermore, ensuring data quality and interoperability remains a critical concern. Manufacturers must invest in robust data management systems and establish standardized protocols to facilitate seamless communication between the various components of the manufacturing process. Addressing these issues can improve process understanding, enable advanced analytics, and support continuous improvement. A prior study found that physics-based simulation models are essential for developing DTs in additive manufacturing, as they allow for accurate predictions of thermal fields and geometric outcomes (Tao et al., 2018). Future research in the field of DTs for additive manufacturing should focus on developing more sophisticated models capable of accurately predicting the behavior of complex materials and processes. This includes exploring advanced simulation systems that incorporate AI/ML to enhance predictive capabilities. In addition, it is important to address ongoing challenges related to data integration and interoperability by developing open standards that facilitate communication across diverse systems and platforms.

## 6 Opportunities, challenges, and future perspectives

### 6.1 Personalized medicine

DTs offer transformative potential in the field of personalized medicine by enabling the development of patient-specific treatment strategies. By creating virtual representations of biological systems that integrate an individual's genetic makeup, medical history, and lifestyle factors, DTs allow researchers and clinicians to simulate various treatment scenarios. This facilitates the identification of the most effective and safest therapeutic interventions tailored to each patient. A notable example is in the field of oncology, where DTs can simulate tumor responses to various chemotherapeutic agents, allowing oncologists to select the most appropriate treatment regimen for individual patients. Such personalization not only improves clinical outcomes but also contributes to more efficient use of healthcare resources. The simulation-based approach supports evidence-based decision-making and reduces reliance on trial-and-error methods in clinical settings. The integration of DTs into healthcare systems enables real-time patient monitoring and predictive analytics. By continuously collecting data from wearable devices and other monitoring tools, they can provide healthcare providers with up-to-date information on a patient's condition. This real-time data can be analyzed to predict potential health issues before they become critical, allowing for timely interventions. For instance, DTs of a patient with chronic heart disease can monitor vital signs and alert healthcare providers to any irregularities, facilitating initiative-taking management of the condition.

### 6.2 Data integration

The effective implementation of DTs depends on the integration and standardization of diverse datasets. In pharmaceutical perspectives, data often comes from various sources, including clinical experiments, electronic health records, and laboratory results. It is challenging to ensure that these datasets are consistent and integrate seamlessly into the framework. Overcoming challenges related to data inter-operability and privacy, complexity, model accuracy, computational resources, validation, limited adoption, and implementation cost remain the critical considerations for future advancements (Kerrouchi et al., 2023). While numerous commercial software platforms such as Siemens Teamcenter *vs.* Dassault BIOVIA exist, the lack of compatible protocols between them presents a practical barrier to building integrated DTs and can lead to issues (Table 3). Network heterogeneity presents another challenge in the deployment of DTs, markedly in industrial settings where various manufacturing tools are utilized (Wang et al., 2022). The diverse nature of these systems can complicate data synchronization and communication between physical entities and their digital counterparts. To address this, it is essential to develop standardized protocols and formats. This standardization facilitates a smooth integration and inter-operability among different systems, enhancing the overall functionality (Bordeleau et al., 2020). However, FDA AI/ML validation draft places emphasis on traceability over standardization.

**Fig. 11:**
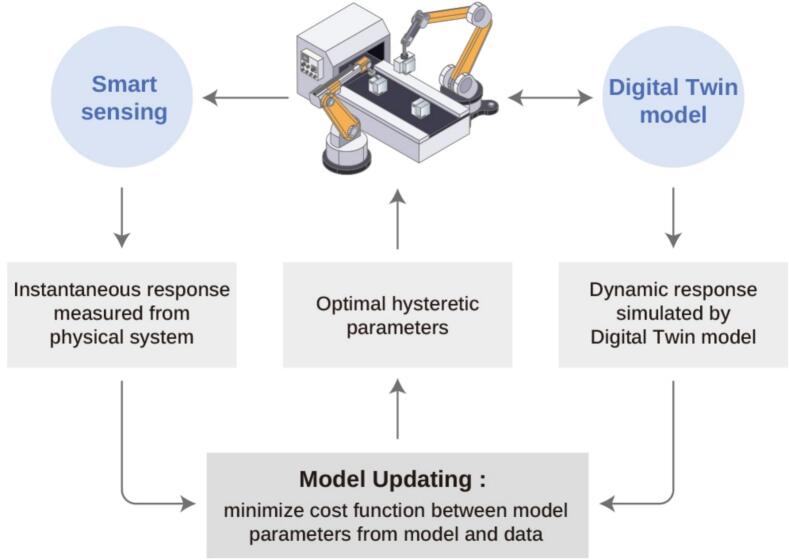
Importance of smart sensing on the model upgrade of Digital Twins with the continuous feeding of responses from physical system and dynamic simulated response from Digital Twins, redrawn from (Soori et al., 2023) under Creative Commons License CC BY 4.0 DEED, permission from Elsevier.

In addition to these data integration issues, technical bottlenecks hinder the practical deployment of DTs. Network-related constraints, such as latency and energy consumption from heterogeneous wireless links and uneven resource distribution, complicate synchronization between physical systems and their DTs. Most biopharma facilities typically use Supervisory Control and Data Acquisition (SCADA) systems with industrial protocols such as OPC UA and Modbus. Fig. 11 illustrates the importance of smart sensing for updating DT models through continuous integration of physical-system responses with corresponding simulated outputs (Fuller et al., 2020). Synchronizing high-frequency sensor data, such as bioreactor readings, with cloud-based DTs imposes substantial computational load, and in continuous manufacturing, maintaining more than 98 % process stability may require millisecond-level adjustments.

### 6.3 Regulatory and ethical considerations

Regulatory approval is another critical consideration in the deployment of DTs, specifically in pharmaceutical applications. Regulatory bodies require rigorous validation and verification of models to ensure their accuracy and reliability (Maharjan et al., 2024b). The process is laborious, posing a barrier to their widespread adoption. Moreover, ethical considerations surrounding data privacy and security must be addressed, especially when dealing with sensitive patient information. Establishing robust cybersecurity measures is essential to protect against data breaches and ensure compliance with regulations such as the Health Insurance Portability and Accountability Act (HIPAA).

As highlighted in Section 4.1, regulatory alignment remains one of the most significant barriers to DT adoption, with extended validation and compliance procedures often hindering their implementation. Hybrid DT models can partially address this issue by improving process understanding, enhancing the quality and consistency of data packages, and providing audit-ready digital records that complement experimental validation. While these models do not currently replace formal validation requirements, they can reduce redundancy and lessen the need for supplementary experiments or resubmissions, thereby alleviating some of the delays commonly encountered during regulatory review. To achieve broader impact, however, formal guidance from agencies such as the FDA and EMA will be essential to define how model-based evidence can be systematically incorporated into regulatory decision-making.

Building on this need for formal guidance, broader regulatory frameworks must also evolve to effectively respond to the complexities introduced by DTs. Current regulations are still largely based on traditional methodologies, creating gaps in the evaluation and application of virtual models. To address these shortcomings, major regulatory agencies should develop explicit guidelines for the assessment and acceptance of DTs in decision-making. Such guidelines should define standards for model accuracy, data integrity, and simulation methodologies, thereby ensuring that digital twin applications remain both scientifically reliable and compliant with established safety and efficacy requirements.

### 6.4 Computational resources and model accuracy

Requirements for computational resources can be substantial to develop and maintain DTs. High-fidelity simulations often demand significant processing power and memory, which can be a limiting factor for organizations. Furthermore, ensuring model accuracy is paramount; inaccuracies can lead to erroneous predictions and potentially harmful clinical decisions. Continuous model validation against real-world data is necessary to maintain their integrity. For instance, Roche's recent case study showed that GDPR-compliant masking to protect anonymity during continuous manufacturing reduced accuracy by nearly 40 %. The frameworks for biologics production, combined with bioreactor sensor feedback and real-time patient-derived data, may be structurally incompatible with DT requirements. There are persistent obstacles in integrating them with other digital technologies, such as modeling information, virtual and augmented reality, IoT, AI, and cloud computing, which is crucial for the digitalization of asset delivery and operations (Eramo et al., 2022). The high complexity of modern industrial systems makes it difficult to integrate multiple relevant DTs across the entire lifecycle of a system, necessitating the use of semantic technologies to enhance cognitive capabilities (Zheng et al., 2022). The development of publicly accessible platforms, such as makeTwin, can significantly enhance the application of DTs in healthcare and education (Tao et al., 2024). From an industrial perspective, DTs improve processes by feeding real-time data back into models, enabling iterative refinement (Fig. 10). These platforms provide healthcare professionals and students with immersive digital experiences, allowing them to explore complex biological systems and treatment scenarios. DTs also hold the potential to optimize the pharmaceutical supply chain. By providing a comprehensive view of the entire supply chain—from raw material sourcing to distribution—DTs can enhance productivity, efficiency, and transparency. They can simulate various supply chain scenarios, allowing companies to identify bottlenecks and optimize planning. This capability is particularly valuable in critical global health crises, where rapid response and adaptability are essential for ensuring the availability of critical medications.

### 6.5 Future trends: AI, ML, and quantum computing

**Fig. 12:**
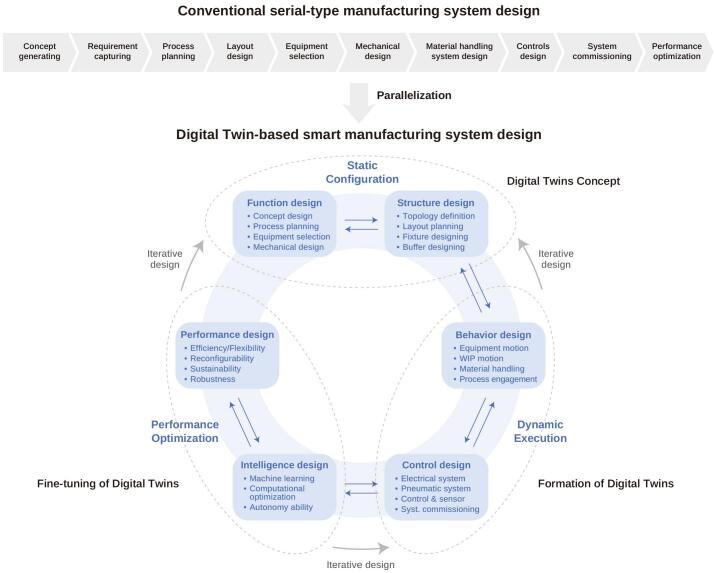
Conventional manufacturing system upgraded to Digital Twin-based smart manufacturing system with implementation of static configuration, dynamic execution, and performance optimization. The process flowsheet is in the order of concept generating → requirement capturing → process planning → layout design → equipment selection → mechanical design → material handling system design → controls design → system commissioning → performance optimization, redrawn from (Soori et al., 2023) under Creative Commons License CC BY 4.0 DEED, permission from Elsevier.

The future development of DTs will be strongly influenced by the integration of advanced technologies such as AI/ML, blockchain, nanotechnology, quantum computing, and dark factory (Nadimpalli et al., 2025; Ünver et al., 2024; Wichter et al., 2024). Among these, AI/ML are particularly critical, as they enable improved predictive accuracy and real-time decision-making by processing large and complex datasets. Blockchain can complement DTs by ensuring data security and integrity (Li et al., 2023b), while nanotechnology offers opportunities for linking DTs with targeted drug delivery systems (Gowda et al., 2022). Quantum computing has the potential to expand DT capabilities by supporting high-dimensional simulations and big data processing beyond the limits of classical computing. Dark factories, powered by DTs, can merge virtual precision with autonomous execution. DTs act as dynamic, data-rich replicas of physical systems, feeding real-time IoT sensor data to predict outcomes (for instance, bioreactor deviations, drug stability) and autonomously optimize processes through closed-loop control. This integration can reduce R&D timeline by replacing up to 60 % of lab experiments (*e.g.*, mRNA vaccine formulation) with *in silico* models, while enabling scalable, personalized therapies (*e.g.*, CAR-T cells) *via* self-adjusting production lines. As DTs evolve into more dynamic and interconnected systems, ensuring cybersecurity and synchronization with physical becomes essential. This is especially relevant in CPS, where virtual models must operate in real time alongside physical processes (Bellavista et al., 2021). The remaining challenges include AI/ML standardization, data synchronization, integration with heterogeneous digital systems, network heterogeneity, and bi-directional communication. Tackling these issues is critical for the successful deployment of intelligent, scalable DT platforms across industries. A transition from conventional manufacturing to DT-enabled smart systems can be supported through structured implementation steps: concept generating → requirement capturing → process planning → layout design → equipment selection → mechanical design → material handling system design → controls design → system commissioning → performance optimization (Fig. 12) (Soori et al., 2023).

## 7 Conclusion

The integration of DTs in drug development is fundamentally reshaping the landscape of smart manufacturing and personalized treatment. Regardless of the obstacles pertaining to data integration, model precision, and regulatory approval, DTs present a significant promise for improving drug development, biopharmaceutical production, and additive manufacturing processes. Future developments will rely on improving simulation accuracy and extending application domains by utilizing AI/ML algorithms and innovative technologies like blockchain, nanotechnology, and dark factory conceptualization. DTs will lead the way for effective, economical, and customized healthcare by tackling critical issues and embracing technological breakthroughs. This will eventually improve patient outcomes and accelerate pharmaceutical innovation.

## CRediT authorship contribution statement

**Ravi Maharjan:** Writing – original draft, Investigation. **Nam Ah. Kim:** Writing – review & editing, Investigation. **Ki Hyun Kim:** Writing – review & editing, Supervision, Funding acquisition, Conceptualization. **Seong Hoon Jeong:** Supervision, Project administration, Funding acquisition, Conceptualization.

## Declaration of competing interest

The authors declare that they have no known competing financial interests or personal relationships that can influence the present work.

## Data Availability

No data was used for the research described in the article.

## Appendix A Supplementary data

Supplementary material Image 1
