# Supplementary material for: Transformative roles of digital twins from drug discovery to continuous manufacturing: pharmaceutical and biopharmaceutical perspectives
Source: Int J Pharm X. 2025 Sep 27;10:100409. doi: 10.1016/j.ijpx.2025.100409 (PMC12516570; doi:10.1016/j.ijpx.2025.100409)
Supplement: Supplementary file 1 — Supplementary material [file mmc1.docx]

**Supplementary Information**

**Transformative roles of digital twins from drug discovery to continuous manufacturing: pharmaceutical and biopharmaceutical perspectives**

Ravi Maharjan^a^, Nam Ah Kim^b,c^, Ki Hyun Kim^b^*, Seong Hoon Jeong^a^*

^a^ College of Pharmacy, Yonsei University, Incheon 21983, Republic of Korea

^b^ College of Pharmacy, Mokpo National University, Jeonnam 58554, Republic of Korea

^c^ Department of Biomedicine, Health & Life Convergence Sciences, BK21 Four, Biomedical and Healthcare Research Institute, Mokpo National University, Jeonnam 58554, Republic of Korea

**Table S1.** Different scope of DTs, along with their respective applications.

| **No.** | **Scope** | **Applications** | **Reference** |
| --- | --- | --- | --- |
| 1. | Automation | - Understand the features, performance, and faults in virtual models - Create model responsive to physical counterpart’s change - Replicate physical assets, activities, and frameworks - Manufacturing is simple and efficient - Reduce production time - Simulate virtual assets before being physically manufactured - Integrate physical, human, and operational data to improve productivity - Forecast, track, and manage inventory in real time - Smart factory to automate and digitalize process - Virtual clone of a tangible element created prior to manufacture - Mimic actual data to understand a product | (Agrawal et al., 2023; Gao et al., 2023; Latsou et al., 2023) |
| 2. | Advanced process | - Predict potential difficulties - Eliminate defects and reduce manufacturing time - Monitor all real-time activities and optimize the action - With a vast volume of data, predict accurate outcome | (Gbadago et al., 2023; Liu et al., 2022) |
| 3. | Building utilization | - Monitor and optimize HVAC, lighting and lower the expense - Detect the issue at an early stage and recommend corrective action - Evaluate the space capacity and intelligent design of the building - Simulate the scenario and identify emergency evacuation routes | (Mourtzis et al., 2021) |
| 4. | Cost effective | - Optimize maintenance, minimize downtime, enable production of goods - DTs and cloud computing reduce design, emulation, and simulation cost - Assess ideas efficiently with small amount of goods/complex machinery - Efficient batch size or optimization | (Qin et al., 2021) |
| 5. | Digitization | - Digital image of a real-world product, process, or equipment - Understand, analyze, and optimize processes through real-time simulation - Use data from sensors linked to a device to run a simulation - AI/ML and sensors grow the concept of DTs | (Wang et al., 2022a) |
| 6. | Data collection | - Collect the data linked with various assets (hardware, operation, analytical) - AI/ML optimizes the performance and self-diagnoses a machine - Decrease the need for manual interference - Virtual models simulate, analyze, predict, and prevent problems in advance - AI/ML, IoT, and data analytics are widely applied in industries | (Gaha et al., 2021; Zotov et al., 2021) |
|  |  | - To implement AI/ML in real scenario, a platform needs to be built |  |
| 7. | Development period | - Minimize the errors throughout the manufacturing process - Discover a bottleneck and reduce the time for the developmental phase - Optimize planning - Support decision-making and choose the best option based on database - Create virtual image of data (production, prior knowledge, data analytics) | (Ladj et al., 2021) |
| 8. | Decision making | - Database collected from data analytics, sensors, and conditions - Influence on how things are created, manufactured, and maintained. - Generate a virtual replica which is continually fed with operational data | (Bhatti et al., 2021; Elahi and Tokaldany, 2021; Niaz et al., 2021) |
| 9. | Error detection | - Interrupt a system to manage unexpected events and improve performance - Improve the risk assessment - Speed up conception of new goods, increase dependability of manufacture - Train a person in hazard conditions/with costly or far-located equipment - Monitor and manage the system’s performance remotely - Along with Industry 4.0, it increases flexibility and efficiency - IoT and physical equipment connected for data transfer and communication - Decrease the current and probable errors in the manufacture setup - Evaluate and simulate the product in a virtual environment - Recreate product life cycle with the improvement in a digital environment - Decrease a risk significantly | (Maïzi and Bendavid, 2021; Warke et al., 2021; Zhou et al., 2021) |
| 10. | Industry performance | - Monitor sensors and relevant data that control the plant’s level of performance - With AI/ML, plan an ideal time for maintenance without affecting production - Promote predictive maintenance across a range of systems - Decrease downtime - Perform self-diagnostics and replace defective parts - Adopt predictive maintenance, reduce downtime due to faulty equipment - Accelerate early installation by simulation, automation, and monitoring - Improve existing operation and shorten maintenance time - With automation, network operation grows to advanced capability | (Maheshwari et al., 2023; Petri et al., 2023; Psarommatis and May, 2023) |
| 11. | Innovation | - Identify the bottleneck in streamlining processes - Spot abnormalities and deviations in the operations promptly - Avoid costly breakdown - Predictive maintenance - Enhance performance with precise virtual image and simulation - Predict failure using model based on risks, events, and software - Reduce downtime and cost, increase equipment efficiency and life - No need to stop the process - Understand a novel approach - Discover setting that produce meaningful consequence | (Alves et al., 2023; Fukawa and Rindfleisch, 2023; Rahmanzadeh et al., 2023) |
| 12. | Information management | - To link sensor with equipment to provide a robust management solution - Generated data are kept in the cloud - Access, detect, and evaluate remotely the physical instrument - Operate remotely to prevent failure, breakdown, and fault - Availability of real-time data and advanced analytics accelerates the project - Dynamic as it gets continually supplied with new data from real world | (Alves et al., 2023; Jafari et al., 2023; Jungmann et al., 2023; Mohammadi et al., 2023) |
| 13. | Mimic physical equipment | - Data management and inter-operable network - Acquire data from devices and products in an existing system - Develop business prospects that optimize all related processes - Replace human labor in identifying broken equipment - Make better operational and strategic decision | (Hu et al., 2023; Wang et al., 2023a) |
|  |  | - Provide visibility into the whole manufacturing process - Virtual tool to evaluate existing instruments, processes, and system - Create virtual images using augmented reality, 3D visualization, modeling - Real-time monitor of physical plants through sensors linked to system - Predict potential breakdowns, downtime, and accidents - Perform diagnostics on virtual plant - Rectify defects with little productivity loss |  |
| 14. | Maintenance | - Predictive analysis - Estimate long-term operation - Initiate data generation from start of equipment design to end of its lifespan - Reduce machine breakdown - Spot potential fault before production by replicate in virtual platform | (van Dinter et al., 2023; Xia and Zou, 2023; Zhong et al., 2023) |
| 15. | Machine's self-awareness | - Detect non-catastrophic problems and align them with another machine - Reduce raw material consumption - Prevent down time and reduce unexpected shutdown - Accomplish the smart factory and Industry 4.0 revolution | (Ma et al., 2023; Vats et al., 2023) |
| 16. | Material handling | - Collects data of the movement pattern, distance, and activity interval - Ensuring efficient resource management - Decrease environmental trace | (Dyck et al., 2023; Saavedra Sueldo et al., 2023; Wang et al., 2024c) |
| 17. | Monitor system | - Monitor system proactively to schedule preventive maintenance - Improve the present goods, process, and service - Discover issues before they exist and forecast future consequences - Address difficulty promptly faster | (Duan et al., 2023; Guo et al., 2023; Jin et al., 2023) |
| 18. | Problem detection | - Detect an issue with the physical counterpart - Identify defects by change in machine's performance and model's behavior - Industry 4.0 includes advanced software system and knowledge transfer - Deploy the insights back to the real world as updated configuration - Co-ordinate and optimize the facility | (Kumbhar et al., 2023; Li et al., 2023c; Xie et al., 2023) |
| 19. | Prediction | - Operate, for instance, weather prediction, historical demand data - Solve problems and deliver performance - Utilize to enhance decision-making process | (Arsiwala et al., 2023; Haghshenas et al., 2023) |
| 20. | Simulation | - Simulate processes that are difficult to predict the outcome - Real-time evaluation reveals undesirable tendencies in process over time - Simulate ad implement in the real world | (Mu et al., 2024; Padovano et al., 2024) |
| 21. | Superior quality | - Build top-quality goods at a low cost - Create a virtual model to evaluate the product under diverse circumstances - Decrease waste and changeover time - Improve product quality - Serve as customer-interactive dynamic supply chain | (Jamshidi and Budak, 2024) |
| 22. | User-friendly service | - Enable complicated digital simulations that are self-reliant - Complexed systems and processes - Identify inconsistencies and design remedial steps to achieve the goals | (Aloqaily et al., 2023; Chen et al., 2024; Liu et al., 2023e) |
| 23. | Virtual replica | - Prior to a physical item, evaluate the virtual replica - Mimic developmental phase, discover potential issues before manufacture - Effect of modification on the results and corrections made to improve - Improve operational steps and lower the engineering cost - Develop IoT for a dependable, efficient, cost-saving application - Collect and analyze operation data to determine condition in real-time | (Gong et al., 2023; Jang et al., 2023) |
